# Spectral, Morphological and Dynamical Analysis of a Holographic Grating Recorded in a Photo-Mobile Composite Polymer Mixture

**DOI:** 10.3390/nano11112925

**Published:** 2021-11-01

**Authors:** Daniele Eugenio Lucchetta, Riccardo Castagna, Gautam Singh, Cristiano Riminesi, Andrea Di Donato

**Affiliations:** 1 Dipartimento di Scienza ed Ingegneria della Materia, dell’Ambiente e Urbanistica (SIMAU), Università Politecnica delle Marche, Via Brecce Bianche, 60131 Ancona, Italy; 2 Consiglio Nazionale delle Ricerche (CNR), Unità di Ricerca presso Terzi (URT-CNR), Università di Camerino (UNICAM), Polo di Chimica, Via Sant’Agostino, 1, 62032 Camerino, Italy; 3 Consiglio Nazionale delle Ricerche (CNR), Institute of Heritage Science, Via Madonna del Piano, 50019 Sesto Fiorentino, Italy; cristiano.riminesi@cnr.it; 4 Department of Applied Physics, Amity Institute of Applied Sciences, Amity University, Uttar Pradesh, Noida 201313, India; gautsingh@gmail.com; 5 Dipartimento di Ingegneria dell’Informazione (DII), Università Politecnica delle Marche, Via Brecce Bianche, 60131 Ancona, Italy

**Keywords:** holography, photomobile polymer, all-optical switching

## Abstract

We report on the morphological, spectral and dynamical characterization of one-dimensional transmission holographic volume phase gratings, whose refractive index contrast and nanometric pitch are dynamically controlled by an incident laser light. The grating is obtained by the photo-polymerization of a recently developed photo-mobile holographic composite polymer material. The observed changes in the refractive index contrast and grating pitch strongly suggest that the reversible all-optical real-time modulation of the obtained diffraction efficiency is induced by nano-fluidics.

## 1. Introduction

All optically addressable holographic gratings have been studied for a long time. They are usually obtained by mixing azo-benzene-based molecules with holographic polymer dispersed liquid crystal (HPDLCs) materials or with different thermotropic nematic liquid crystals (NLCs) [1,2,3,4,5,6,7,8,9,10,11,12]. Those mixtures are sensitive to the polarization state of an impinging light, which affects the chemical configuration of the active azo-dye molecules present in the mixture. Those changes lead to a different orientation and alignment of the liquid crystal molecules in the system, resulting in detectable modifications of the physical parameters characterizing the transmission or reflection properties of the grating. An all-optical switching device based on a one-dimensional transmission grating recorded in a SU-8 polymeric substrate infiltrated with a dye-doped liquid crystal was also studied. The dynamical properties of this highly nonlinear mixture were successfully used to modulate the diffraction proprieties of the grating by a pump beam when a static electric field was applied [11]. More recently, azo-benzene-based photo-mobile polymer thin films were processed by polarized light to generate and orient surface gratings [13]. A holographic mixture allows us to optically record holographic structures. After the recording process, some physical parameters of the writing light source, such as its polarization state, writing wavelength and angle of incidence, can be retrieved by investigating the optical properties of the reconstructed beam [14]. In this work, we focus on a novel holographic transmission phase grating whose efficiency is all-optically controllable through reversible light-induced changes in the refractive index modulation of a photo-mobile polymer in which the grating is recorded [15]. Our syrup is based on a recently developed composite polymer mixture which is holographic and photo-mobile at the same time [15,16,17]. The addition of a synergic photo-initiator system allows polymerization in the visible range at λ= 457.9 nm. Accordingly, we obtain holographic transmission volume gratings (*d*∼ 75 μm and a pitch Λ∼ 500 nm) whose diffraction efficiency is all-optically modulated by means of an incident pumping laser at 457.9 nm. The contributions due to light-induced refractive index modulation and pitch are separately studied. The morphological properties of the grating are also investigated by using an Atomic Force Microscope (AFM).

## 2. Materials and Methods

### 2.1. Materials

In this work, 2,3-bornanedione (CQ), tri-phenyl-o-methane-triglicidyl ether (TPMTGE), dipentaerythritol monohydroxypentaacrylate (DPMHPA), lead (IV) tetra-acetate, 4-amino-phenol (4-AP), phenyl-(2,4,6-trimethylbenzoyl) phosphoryl]-(2,4,6-trimethylphenyl)methanone (PTPTM) and N-Vinylbutyrolactam (NVBL) came from Sigma Aldrich (Milan, Italy); lead(II) oxide (PbO2) was freshly prepared by the hydrolysis of lead(IV) acetate.

### 2.2. Holographic Mixture Preparation

The mixture was prepared starting from the recipe indicated in [16] to which the epoxide-monomer TPMTGE and the photo-initiator 2,3-bornane-dione (CQ) were then added. Monomers were TPMTGE:DPMHPA:NVBL (0.1:2:5, molar ratio), and the photo-initiators were CQ:PTPTM (0.2:0.1 molar ratio); 4-AP:lead (II) oxide (1:0.5 molar ratio). The reaction between NVBL, 4-AP and lead (II) oxide was conducted in aerobic conditions at room temperature under magnetic stirring; after one week, the reaction was left at rest at room temperature in darkness for another week. The precipitate (accumulated in the reaction) was carefully removed from the reaction environment, and the reaction medium was filtered to remove undesired residues of lead oxide. Separately, DPMPHA was mixed with TPMTGE. For this, TPMTGE was heated at the temperature of 90 ${}^{\circ }$C. At this temperature, the TPMTGE was in a low viscous form and could be easily added in a small bottle to be mixed with DPMPHA. The mixture was left under mechanical stirring until a pale yellow color was obtained. The obtained mixture was then added to the previous one obtained following the recipe indicated in [16], and the complete final mixture was blended under magnetic stirring for a further 36 h in the dark. The initiators were finally added to the system, which was left under magnetic stirring for 24 h.

### 2.3. Holographic Set-Up

**Figure 1:**
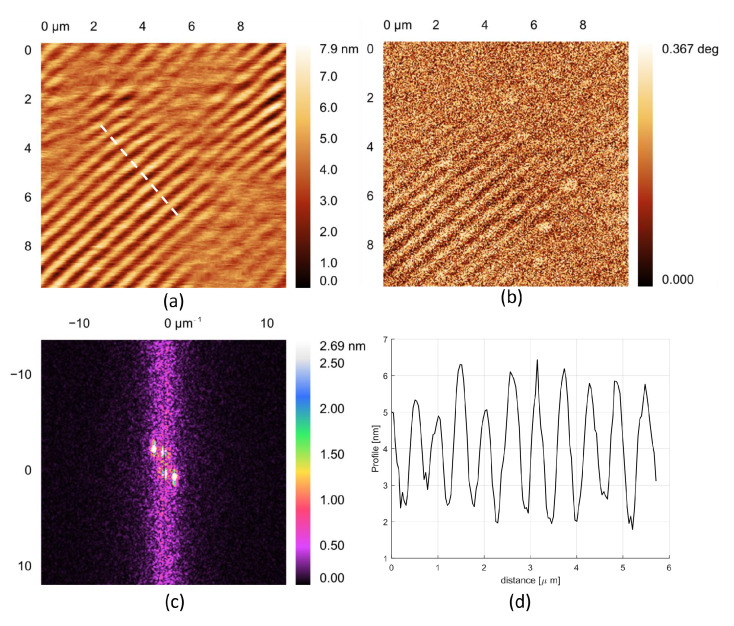
(**a**) Surface topography obtained by means of Atomic Force Microscopy working in non-contact mode at a resonance frequency of about 150 kHz, through a tip with radius of curvature of about 20 nm. (**b**) Phase image obtained by using the Phase Contrast Microscopy technique to highlight the surface spatial modulation of the refractive index. (**c**) Amplitude of the Fast Fourier Transform of topography. The peak positions are located at a spatial frequency corresponding to pitch of about 520 nm. (**d**) Profile taken along the dotted white line, as highlighted in topography.

Our standard cell was made by two microscope glasses separated by two 76 μm thick Mylar stripes. The cell was heated at around 60 °C and the mixture was forced to enter by capillarity. After that, the sample, left for one hour at room temperature on the sample holder, was irradiated by two interfering continuum s-polarized laser beams at 457.9 nm (see setup A in the Supplementary Materials). During the photo-polymerization, a phase separation process occurred between polymerized and unpolymerized regions of the mixture and, as a result, a one-dimensional (1D) holographic grating was permanently recorded inside the interfering region. This region had a diameter of 5 mm. The used writing power was 150 mW per beam. A low-power 632.8 nm He–Ne laser positioned at the Bragg diffraction angle was used to detect the grating formation. After three minutes, the grating was completely recorded. To ensure a complete photo-polymerization of the spot area was performed, and the total irradiation time was set at 10 min. The spectral angular analysis of the diffracted wavelengths was performed by illuminating the grating, placed on a motorized goniometer, through an optical fiber connected to an incoherent Xe light source emitting wavelengths in the range 350–1000 nm. The spectra of the transmitted light were detected using a real-time spectrometer for each value of the incident angle (see experimental setup B in the Supplementary Materials). Each angle was a Bragg angle for the corresponding narrow range of diffracted wavelengths. Each spectrum indeed showed a quite narrow peak centered around the main diffracted wavelength. The height of the peak, after a proper calibration of the spectrometer, directly gave the value of the diffraction efficiency of our diffracted signal. In this way, it was possible to obtain a complete set of experimental points by directly measuring the value of diffraction efficiency as function of the wavelength or Bragg angle [18,19]. The dynamical characterization of our samples was conducted by illuminating the grating with white light at a fixed Bragg angle corresponding to the maximum measured diffraction efficiency value. Measurements were performed by pumping the sample with a blue DPSS laser operating at 457.9 nm and acquiring the spectra as function of time by using a real-time spectrometer (see experimental setup C in the Supplementary Materials). After the spectral and dynamical characterizations, the microscope slides forming the sandwich-like cell were detached from each other, leaving the polymerized spot exposed to open air and anchored to one side of the slide. In this way, it was possible to perform contrast-phase imaging through an Atomic Force Microscope, which was able to characterize at the nanoscale the topography and refractive index modulation of the grating. In Figure 1, we report the collected images and data acquired with a scanning window of 10 μm × 10 μm, proving the presence of the written phase grating.

## 3. Results and Discussions

**Figure 2:**
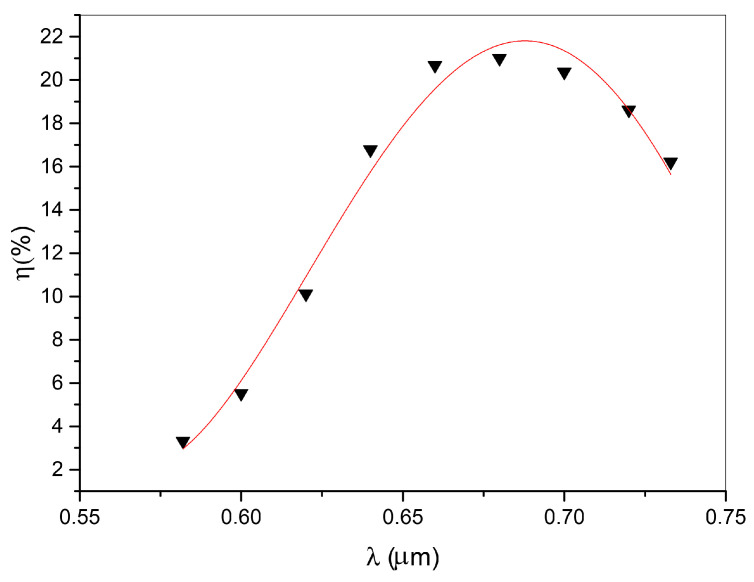
Maximum diffraction efficiency η as a function of wavelength. The continuous line represents the theoretical data fit obtained by using Equation (1).

The results obtained from the spectral analysis are reported in Figure 2, showing the diffraction efficiency for the s reading polarization at different wavelengths. The continuous line represents the theoretical data fit obtained by using Equation (1), in which the Bragg angle is written as function of the corresponding wavelength.

Values of diffraction efficiency varied approximately from 3% to 20% in the whole set of our investigated samples, showing a FWHF (full width at half maximum) of the central peak of ∼0.03 rad. The well-known expression of the diffraction efficiency derived by the theory [20] was used to fit the experimental data. Accordingly, the diffraction efficiency of a one-dimensional transmission phase grating can be written as

(1) $$\eta \left(\nu ,\xi \right)=e^{-\frac{\alpha d}{\cos\theta }}\frac{{sin\left(\sqrt{\left(\nu^{2}+\xi^{2}\right)}\right)}^{2}}{1+\frac{\xi^{2}}{\nu^{2}}}$$

with coupling and detuning parameters, respectively, defined as

(2) $$\nu =\frac{\pi \cdot \delta n\cdot d}{\lambda \cdot cos\theta }$$

(3) $$\xi =\Delta \theta \cdot \beta \cdot d\cdot sin\theta_{0}$$

where δn is the induced refractive index variation, *d* is the grating thickness, λ is the reading wavelength in the free space, θ is the angle of incidence, $\theta_{0}$ is the Bragg angle, α is the distributed absorption coefficient, *n* is the average refractive index of the medium and β=2πn/λ.

The agreement between the theoretical expression reported in Equation (1) and the experimental data was excellent and allowed the determination of the grating refractive index modulation $\delta n∼2.3\times 10^{-3}$, pitch Λ = 512.8 nm and thickness *d* = 77.98 μm. The data were corrected for angle and polarization-dependent Fresnel refraction. The parameter Δθ in Equation (3) describes the de-phasing term that appears when λ or θ are varied. Their connection is shown by the following relation, in which the grating period Λ appears explicitly:

(4) $$\Delta \theta =\frac{2\pi }{\Lambda }\cdot \sin\theta -{\left(\frac{2\pi }{\Lambda }\right)}^{2}\frac{\lambda }{4\cdot \pi \cdot n}$$

Under the Bragg condition Δθ=0, the Equation (4) takes the well-known expression:

(5) $$\theta_{0}\left(\lambda \right)=\mathrm{asin}\left(\frac{\lambda }{2\cdot \Lambda \cdot n}\right)$$

which leads to a new expression for the diffraction efficiency:

(6) $$\eta \left(\lambda ,\delta n\right)=e^{-\;\;\frac{2\alpha d}{\cos\theta_{0}\left(\lambda \right)}}\;\sin^{2}\left(\frac{\pi \cdot \delta n\cdot d}{\lambda \cos\theta_{0}\left(\lambda \right)}\right)$$

Assuming an absorption coefficient α independent of the working wavelength λ and the intensity of the pumping laser applied to the sample, we can derive from Equation (6) the refractive index deviation δn as a function of the diffraction efficiency at the measured wavelength λ, as follows:

(7) $$\delta n\;=\;\frac{\lambda \cos\theta_{0}\left(\lambda \right)}{\pi \cdot d}\mathrm{asin}\left(e^{\;\frac{\alpha d}{\cos\theta_{0}\left(\lambda \right)}}\sqrt{\eta (\lambda )}\right)$$

**Figure 3:**
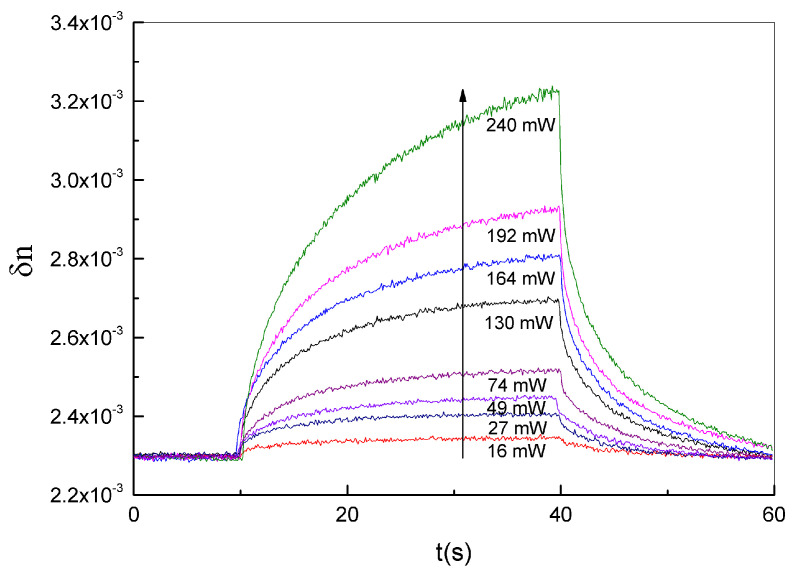
Change of the refractive index contrast δn as function of time for different irradiation power levels from 16 mW to 240 mW @ λ = 457 nm.

Equation (7) defines the non-linear analytical relationship between the measured efficiency and the refractive index contrast. This clearly highlights how an increase of the diffraction efficiency under pumping illumination can be related to changes in the refractive index modulation δn. The above equation is exploited to derive the dynamical behavior of the refractive index deviation for different power levels of the pumping laser, as reported in Figure 3.

**Figure 4:**
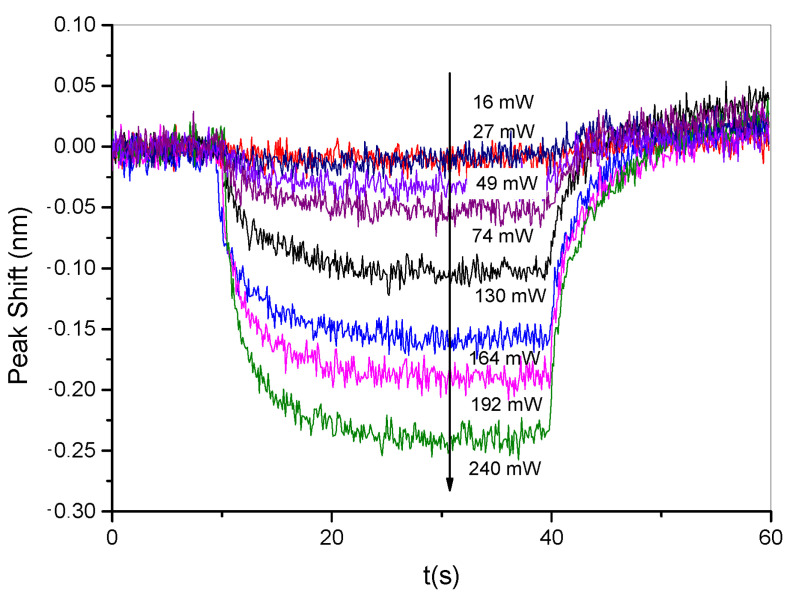
Peak shift of the recorded spectrum as a function of time for different irradiation power levels from 16 mW to 240 mW at λ = 457 nm.

In particular, in order to improve the sensitivity of measuring the changes of refractive index modulation due to the pumping process (see setup C in Supplementary Materials), each transmitted spectrum acquired at the Bragg angle, (corresponding to the maximum diffraction efficiency value reported in Figure 2), was fitted with a Gaussian distribution. From each fitting, it was possible to derive the amplitude and position of the spectral peak. After a proper data normalization, which considers the grating response at rest without external pumping, the diffraction efficiency was computed at each peak position and used to derive, according to Equation (7), the refractive index deviation δn. Figure 3 shows a reversible increase of δn from $2.3\times 10^{-3}$ to $3.2\times 10^{-3}$ during the irradiation time (around 30 s) and a relaxation towards its initial value after the pumping laser is switched-off. Dynamical behavior is directly related to the dynamical variation of the grating diffraction efficiency. The collected fitted spectra, however, can provide additional insights into the behavior of the holographic grating during the irradiation time. As reported in Figure 4, a slight shift of the spectral peak towards lower wavelengths is highlighted when the pumping power is increased. This behavior, in combination with Equations (5) and (6), can confirm the theory according to which—since we are working at the Bragg angle—the modulation of refractive index plays the main role in the dynamical behavior of the grating. In fact, as the refractive index contrast varies, the condition of maximum efficiency defined by Equation (6) is satisfied for a different wavelength of the recorded spectrum.

The results reported in Figure 3 illustrate the increase of the refractive index contrast under irradiation, whereas those reported in Figure 4 show that the pitch Λ of the polymerized rigid grating is subject to a very negligible deformation under irradiation. Both results strongly suggest that the unpolymerized part of the holographic mixture in the grating tends to escape from the irradiated area. In these conditions, the grating refractive index changes, while the morphological properties of the grating are substantially preserved.

## 4. Conclusions

In conclusion, we report on the morphological, spectral and dynamical analysis of one-dimensional holographic transmission phase grating, written on a novel composite holographic photo-mobile material. The experimental data show the presence of a volume phase grating with a pitch Λ of 512.8 nm and a maximum diffraction efficiency of about 22% at around 0.7 μm. The features of the novel holographic polymer mixture make the physical properties of the grating all-optically switchable and reversible by an external impinging laser light. An explanation of the working mechanism, based on light-induced changes of the refractive index contrast of the grating, is provided. This technology can play a relevant role in the growing fields of dynamic holography, augmented reality, 3D vision and free-space optical communications.

## Data Availability

Data are available from the authors.

## Supplementary Materials

The following are available online at https://www.mdpi.com/article/10.3390/nano11112925/s1, Figure S1: The complete description of the experimental set-ups.
