# Supplementary material for: Spectral, Morphological and Dynamical Analysis of a Holographic Grating Recorded in a Photo-Mobile Composite Polymer Mixture
Source: Nanomaterials (Basel). 2021 Nov 1;11(11):2925. doi: 10.3390/nano11112925 (PMC8622520; doi:10.3390/nano11112925)
Supplement: Supplementary file 1 [file nanomaterials-11-02925-s001.zip › nanomaterials-1426112-supplementary.pdf]

Supplementary Material

Spectral, Morphological and Dynamical Analysis of a Holographic Grating Recorded in a Photo-mobile Composite Polymer Mixture

Daniele Eugenio Lucchetta [1,*,†], Riccardo Castagna [2,3,*,†], Gautam Singh [4], Cristiano Riminesi [3] and Andrea Di Donato [5,*,†]

[1] Dipartimento di Scienza ed Ingegneria della Materia, dell'Ambiente ed Urbanistica (SIMAU), Università Politecnica delle Marche, Via Brecce Bianche, 60131 Ancona, Italy
[2] Consiglio Nazionale delle Ricerche (CNR), Unità di Ricerca presso Terzi (URT-CNR), Università di Camerino (UNICAM), Polo di Chimica, Via Sant'Agostino, 1, 62032 Camerino, Italy
[3] Consiglio Nazionale delle Ricerche (CNR), Institute of Heritage Science, Via Madonna del Piano, 50019 Sesto Fiorentino, Italy; cristiano.riminesi@cnr.it
[4] Department of Applied Physics, Amity Institute of Applied Sciences, Amity University, Uttar Pradesh, Noida 201313, India; gautsingh@gmail.com
[5] Dipartimento di Ingegneria dell'Informazione (DII), Università Politecnica delle Marche , Via Brecce Bianche, 60131 Ancona, Italy
* Correspondence: d.e.lucchetta@staff.univpm.it (D.E.L.); riccardo.castagna@cnr.it (R.C.); a.didonato@staff.univpm.it (A.D.D.)
† These authors contributed equally to this work.

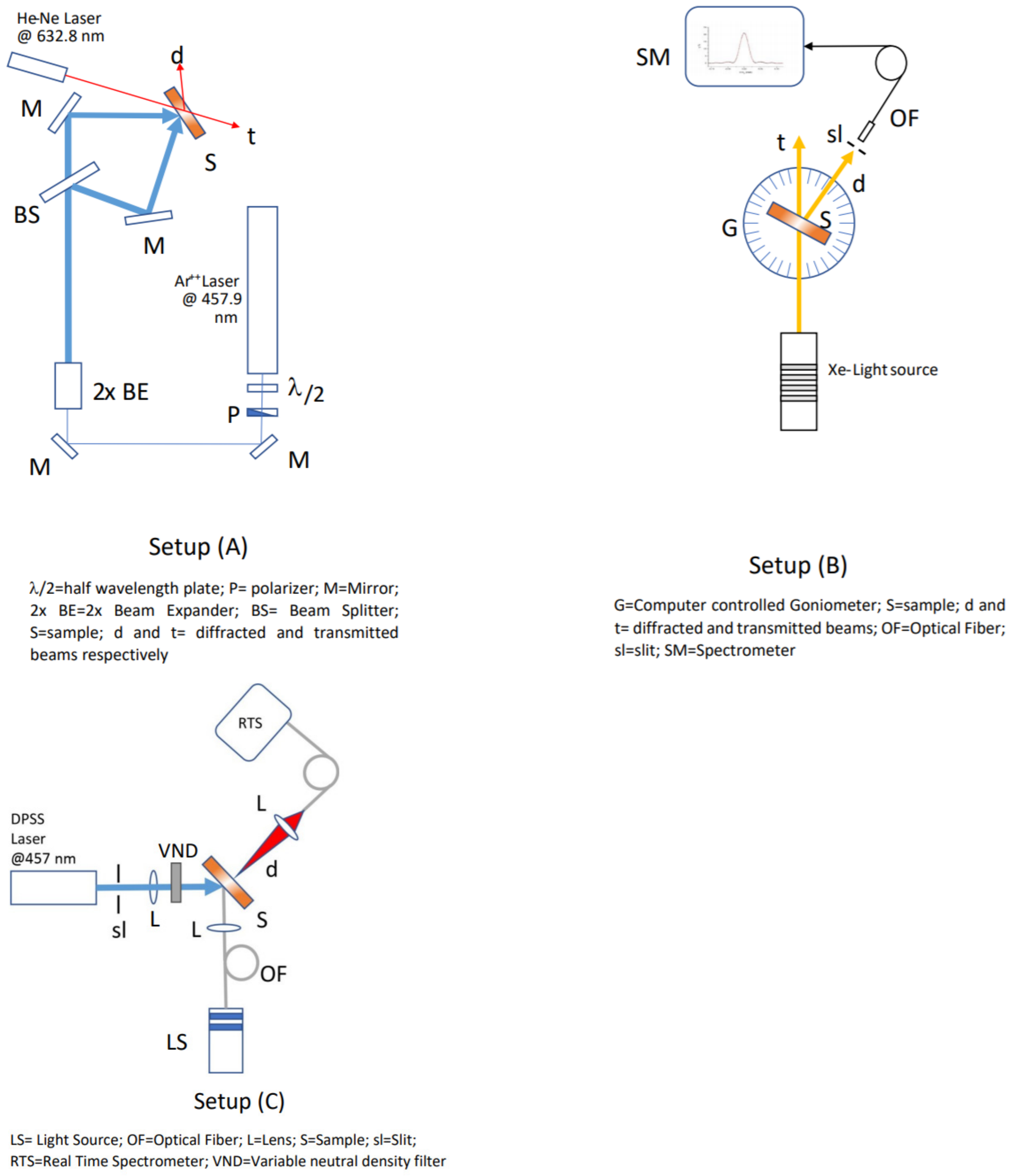

Figure S1. The complete description of the experimental set-ups.
